# Roles of cuproptosis in central nervous system tumors: from molecular mechanisms to therapeutic prospects

**DOI:** 10.3389/fcell.2026.1914214

**Published:** 2026-09-15

**Authors:** Pengpeng Li, Yangyang Gao, Wei Liu

**Affiliations:** Department of Neurosurgery, Xi’an Aerospace Hospital of Northwest University, Shanxi, China

**Keywords:** central nervous system tumors, copper metabolism, cuproptosis, cuproptosis resistance, FDX1

## Abstract

Cuproptosis is a form of programmed cell death reported in 2022, characterized by copper ions directly binding to mitochondrial lipoylated proteins, inducing aberrant protein aggregation and destabilizing iron-sulfur cluster proteins, ultimately leading to cell death. Distinct from apoptosis, ferroptosis, and other known death modalities, cuproptosis does not rely on classical caspase pathways but exerts its cytotoxic effects by interfering with mitochondrial metabolism. Copper, an essential trace element for humans, maintains its homeostasis through precise regulation by transporters such as CTR1 (copper transporter 1), ATP7A (ATPase copper transporting alpha), and ATP7B (ATPase copper transporting beta). Dysregulated copper metabolism is closely associated with tumor initiation and progression. The central nervous system, being one of the organs with the highest copper content, exhibits heightened sensitivity to copper homeostasis imbalance. During metabolic reprogramming, central nervous system tumors such as glioma and medulloblastoma display enhanced copper dependency, rendering them potential targets for cuproptosis-inducing agents. However, tumor cells can develop resistance to cuproptosis through multiple mechanisms, including upregulation of ATP7A/ATP7B to promote copper efflux, downregulation of FDX1 (ferredoxin 1) and DLAT (dihydrolipoamide S-acetyltransferase) to reduce molecular sensitivity, activation of antioxidant systems such as glutathione, and metabolic switching toward glycolysis. Although several reviews have summarized cuproptosis mechanisms, a comprehensive synthesis focusing specifically on central nervous system tumors remains lacking. This review aimed to systematically examine the molecular basis of copper metabolism and cuproptosis, research progress in various central nervous system tumors, resistance mechanisms and counterstrategies, and cuproptosis-targeted therapeutic approaches. By bridging mechanistic insights with translational challenges unique to neuro-oncology, this review aimed to provide a theoretical foundation for the clinical application of cuproptosis-based therapies and to guide future research directions. Current evidence demonstrates that copper ionophores such as elesclomol and disulfiram, copper-based nanomaterials, and combination strategies with immunotherapy or radiotherapy exhibit promising antitumor efficacy in central nervous system tumor models. Meanwhile, challenges including blood-brain barrier delivery efficiency, normal neuron protection, and elucidation of resistance mechanisms remain major hurdles for clinical translation. The findings of this review could provide a theoretical foundation for the translational application of cuproptosis in neuro-oncology and guide future research directions.

## 1 Introduction

Central nervous system tumors, particularly glioblastoma, represent one of the adult malignancies with the poorest prognosis (Sahm et al., 2023). Even with comprehensive treatment incorporating maximal safe resection, radiotherapy, and temozolomide chemotherapy, the median survival of patients remains less than 2 years. The core barriers to treatment failure include the blood-brain barrier limiting drug entry into the brain, tumor heterogeneity and glioma stem cell-mediated therapeutic resistance, and acquired chemotherapy resistance (Tang et al., 2025). These challenges compel researchers to continuously explore novel therapeutic strategies.

Cuproptosis is a novel form of cell death first defined by Tsvetkov et al. (2022) Its pathogenic process can be summarized as follows. Three key enzymes in the tricarboxylic acid cycle, namely, pyruvate dehydrogenase, α-ketoglutarate dehydrogenase, and branched-chain ketoacid dehydrogenase, depend on lipoylation for their activity. Under copper overload conditions, copper directly binds to these lipoylated proteins, inducing their aberrant aggregation and functional loss, thereby blocking the tricarboxylic acid cycle and disrupting energy metabolism (Liu W. et al., 2024). Simultaneously, copper ions attack mitochondrial iron-sulfur cluster proteins such as ferredoxin FDX1 and lipoic acid synthase LIAS (lipoic acid synthase), leading to decreased stability of these proteins and further exacerbating metabolic dysfunction (Tsvetkov et al., 2022). This mechanism does not depend on classical apoptotic effector molecules such as caspases, offering the potential to bypass common apoptotic resistance pathways in tumor cells (Yang et al., 2025a). Moreover, cuproptosis directly targets mitochondrial metabolism, and mitochondrial dysfunction is a prominent feature of tumor cells, providing a basis for selective tumor cell killing. Although several review articles have summarized the molecular mechanisms of cuproptosis and its roles in various cancers, a comprehensive synthesis specifically addressing central nervous system tumors has not been available (Markouli et al., 2025; Xu et al., 2026). In particular, published reviews have not systematically integrated the unique copper metabolic environment of the brain, the distinct sensitivity and resistance profiles across different CNS tumor subtypes, or the emerging nanomaterial-based delivery strategies (Chen et al., 2025a). Furthermore, recent studies on glioma stem cell resistance mechanisms and copper-based combination therapies have not been adequately covered in existing reviews (Xu et al., 2026), highlighting the need for an up-to-date and focused synthesis.

The central nervous system has a special connection with copper metabolism. The brain is one of the organs with the highest copper content in the human body, and copper ions participate in important physiological processes including myelination, synaptic plasticity, and neurotransmitter synthesis (Locatelli and Farina, 2025). Glioma cells are highly dependent on mitochondrial function during rapid proliferation, and copper, as a key cofactor for mitochondrial respiratory chain complex IV, results in higher copper demand in glioma cells and frequent aberrant activation of copper metabolic pathways (Markouli et al., 2025). This dependency renders glioma cells more sensitive to cuproptosis inducers, providing a theoretical foundation for targeted therapy (Gao et al., 2025).

Of course, tumor cells do not remain passive. With advancing research, it has become evident that tumor cells can develop resistance to cuproptosis through multiple mechanisms, including upregulation of copper efflux proteins, downregulation of key effector molecules, activation of antioxidant systems, and metabolic reprogramming (Li et al., 2026). Overcoming these resistance mechanisms is key to advancing the clinical translation of cuproptosis-based therapies.

This review article aimed to summarize the molecular basis of copper metabolism, systematically dissect the core mechanisms of cuproptosis, focus on research progress in central nervous system tumors, analyze cuproptosis resistance mechanisms and countermeasures, and summarize current challenges and future directions. The findings of this review could provide a theoretical foundation for the translational application of cuproptosis in neuro-oncology and guide future research directions.

## 2 Molecular basis of copper metabolism and specificity of the central nervous system

Copper serves as a cofactor for multiple key enzymes, and its physiological functions are relatively well understood. What truly deserves further inquiry is why central nervous system tumors are so sensitive to copper metabolic abnormalities. The brain is one of the organs with the highest copper content in the human body (Zheng and Monnot, 2012), with the highest concentrations found in regions such as the substantia nigra, locus coeruleus, and basal ganglia, which happen to be susceptible sites for neurodegenerative diseases like Parkinson’s disease (Wang et al., 2025). This phenomenon has long been used to explain the pathogenesis of neurodegenerative diseases, but it simultaneously raises a question less frequently discussed in neuro-oncology: have tumor cells originating from these high-copper regions adapted to the high-copper environment, thereby developing a unique copper metabolic dependency?

The maintenance of intracellular copper homeostasis relies on a sophisticated transport system. Copper ions primarily enter cells through the high-affinity transporter CTR1 (SLC31A1), whose expression is regulated by negative feedback from intracellular copper levels (We et al., 2025). Once in the cytoplasm, copper ions are guided to specific subcellular localizations by different copper chaperones. ATOX1 (antioxidant one copper chaperone) delivers copper to ATP7A and ATP7B in the trans-Golgi network for activation of copper-dependent enzymes in the secretory pathway (Wang et al., 2026); CCS (copper chaperone for superoxide dismutase) delivers copper to superoxide dismutase 1 (Suzuki et al., 2013); COX17 (cytochrome c oxidase copper chaperone) delivers copper to cytochrome c oxidase in mitochondria (Maung et al., 2021). Copper efflux is primarily mediated by ATP7A and ATP7B, which pump copper ions out of the cell or transport them into vesicles when copper is excessive (Hartwig et al., 2021). ATP7A mutations cause Menkes disease, while ATP7B mutations cause Wilson disease (Yang et al., 2024). Both genetic disorders fully illustrate the importance of copper homeostasis for the nervous system.

However, glioma research is reshaping this picture. CTR1 is significantly overexpressed in glioma, with expression levels increasing with WHO grade, and high expression correlates with shortened overall survival and an immunosuppressive microenvironment (Wang et al., 2023). ATP7A overexpression is associated with chemotherapy resistance (Yuan et al., 2026). Based on these observations, concurrent upregulation of both copper uptake (CTR1) and efflux (ATP7A) systems in glioma, we propose a “copper tension” hypothesis, in which glioma cells actively increase copper uptake to meet proliferative demands while simultaneously upregulating copper efflux to prevent copper overload-induced toxicity. This regulatory pattern suggests that glioma cells maintain copper levels within an optimal window that supports proliferation without triggering cell death. However, it should be noted that this hypothesis is primarily derived from correlative expression data and has not been directly validated by quantitative measurements of intracellular free copper or total copper levels in glioma cells versus normal counterparts. Direct experimental evidence, such as comparative analysis of labile copper pools and total copper content using copper-specific probes or inductively coupled plasma mass spectrometry, is currently lacking and represents a critical direction for future investigation. If this hypothesis holds true, strategies targeting cuproptosis need to consider how to disrupt this delicate balance.

The association between copper metabolism and tumor progression has been extensively studied in peripheral tumors. For example, copper promotes angiogenesis by activating vascular endothelial growth factor, initiates pro-proliferative signaling pathways such as PI3K/AKT and MAPK, promotes epithelial-mesenchymal transition, and participates in metabolic reprogramming (Guo et al., 2025a). However, whether these mechanisms directly apply to central nervous system tumors requires cautious validation. The presence of the blood-brain barrier makes the significance of angiogenesis in brain tumors different from that in peripheral tumors. Furthermore, the division of labor in copper metabolism between neurons and astrocytes, with neurons highly expressing ATP7A and superoxide dismutase one and astrocytes highly expressing metallothionein (Hossain et al., 2024), further increases the complexity of copper metabolic regulation in brain tumors. It remains unclear to what extent glioma cells retain the copper metabolic features of their cell types of origin and to what extent they have undergone tumor-specific reprogramming. This knowledge gap represents a critical bottleneck currently facing the field.

## 3 Molecular mechanisms and regulatory networks of cuproptosis

### 3.1 Core molecules and pathways

The core molecular components of cuproptosis have been largely identified, mainly including FDX1, DLAT, LIAS, and various iron-sulfur cluster proteins. Similar to the roles of the caspase family in apoptosis or GPX4 (glutathione peroxidase 4) in ferroptosis, FDX1 plays an irreplaceable initiator role in cuproptosis (Tsvetkov et al., 2019). FDX1 has dual functions. It reduces divalent copper ions to monovalent copper ions, the latter being the active form that binds to lipoylated proteins. It also regulates the synthesis of lipoylated proteins in mitochondria. FDX1 expression levels show a clear positive correlation with cuproptosis sensitivity, and FDX1-deficient cells are almost completely resistant to cuproptosis induction (Tsvetkov et al., 2022). This finding has direct clinical translational significance, as different tumor cells exhibit considerable variation in cuproptosis sensitivity, and FDX1 (Figure 1) expression levels can at least partially explain this variation. Transcription factors including NFKB1, p53, and MYC are known to participate in FDX1 expression regulation (Guowei et al., 2023). In glioma, FDX1 is directly regulated by NFKB1 (nuclear factor kappa B subunit 1), and glioma cells with high FDX1 expression are more sensitive to elesclomol, with FDX1 knockdown significantly diminishing sensitivity (Wu et al., 2025a). This suggests that FDX1 is not only a key molecule in mechanistic studies but may also serve as a biomarker for patient stratification.

**FIGURE 1:**
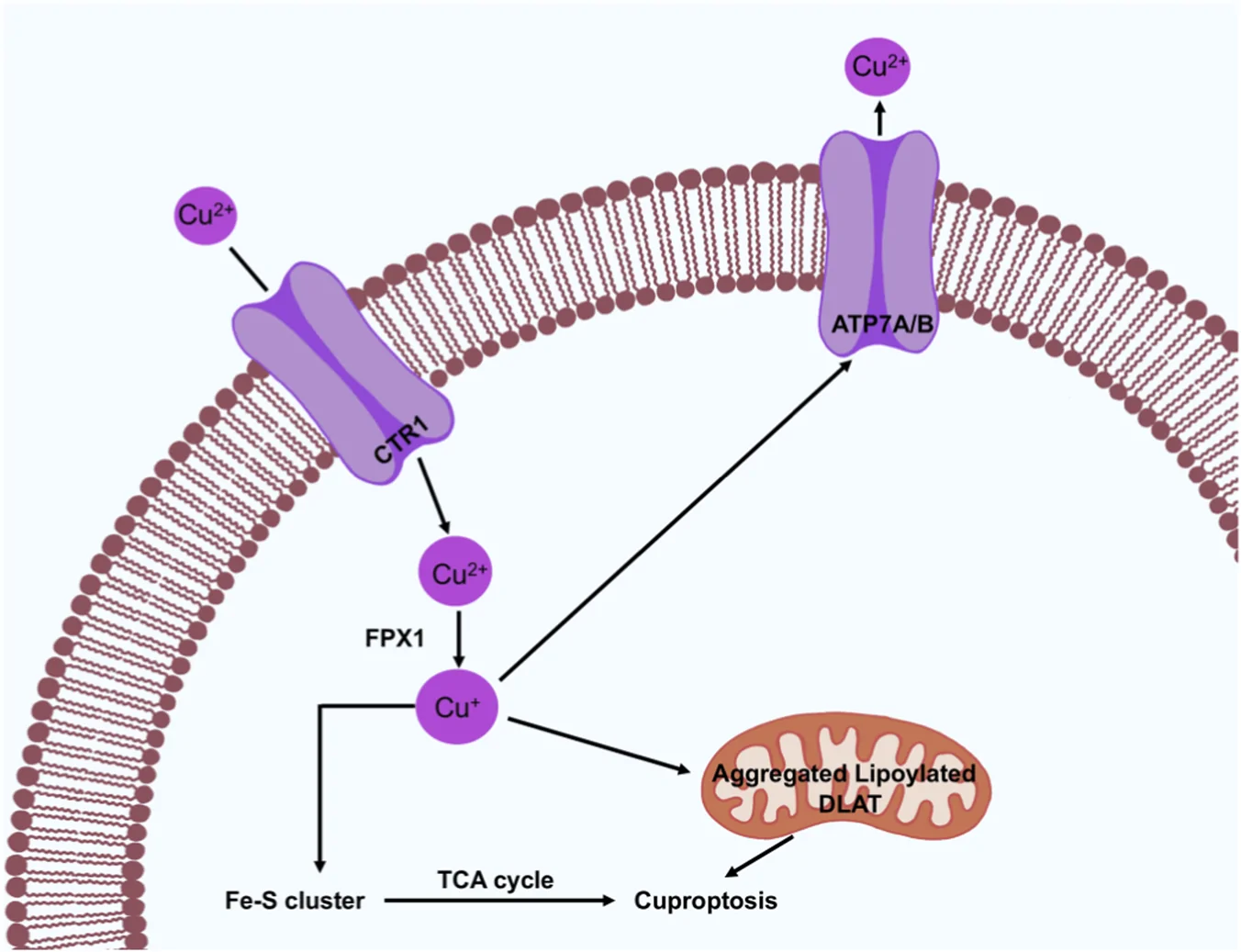
Molecular mechanisms of cuproptosis. Excess copper enters cells via CTR1 and accumulates in mitochondria, where FDX1 reduces Cu^2+^ to Cu^+^. Monovalent copper binds lipoylated DLAT, inducing aberrant oligomerization that disrupts the pyruvate dehydrogenase complex and blocks the TCA cycle. Concurrently, copper destabilizes iron-sulfur cluster proteins (including FDX1 and LIAS), creating a self-amplifying feedback loop of mitochondrial dysfunction that triggers irreversible, caspase-independent cell death. Abbreviations: CTR1, copper transporter 1; FDX1, ferredoxin 1; DLAT, dihydrolipoamide S-acetyltransferase; TCA, tricarboxylic acid.

DLAT is a core effector molecule in cuproptosis execution. As the E2 subunit of the pyruvate dehydrogenase complex, the three lipoylation sites on DLAT can directly bind to reduced monovalent copper ions, inducing aberrant oligomerization of DLAT and formation of protein aggregates, thereby interfering with mitochondrial function (Tsvetkov et al., 2022). Subsequent studies have further confirmed that this aggregation is a specific event in cuproptosis and does not occur in other death modalities such as apoptosis or ferroptosis, making lipoylated DLAT oligomers widely used as a characteristic molecular marker of cuproptosis (Zhu et al., 2024a; Wu et al., 2025b).

LIAS, as the key enzyme responsible for DLAT lipoylation, functions downstream of FDX1. Dreishpoon et al. demonstrated that FDX1 regulates cellular protein lipoylation by directly binding to LIAS, which catalyzes the lipoylation of DLAT and other TCA cycle enzymes (Dreishpoon et al., 2023). Genetic knockout of LIAS significantly inhibits cuproptosis induced by elesclomol-copper and disulfiram-copper, confirming LIAS as an indispensable upstream regulatory molecule for cuproptosis (Liu et al., 2025a).

Mechanistically, the enzymatic activity of LIAS depends on the integrity of its [4Fe-4S] cluster. When this cluster is damaged, LIAS function is lost, leading to decreased DLAT lipoylation levels and altered cellular sensitivity to cuproptosis. Thus, LIAS expression levels indirectly determine cellular sensitivity to cuproptosis by regulating the extent of DLAT lipoylation.

Iron-sulfur cluster proteins represent another important target of cuproptosis. FDX1, LIAS, and multiple proteins in the respiratory chain complex all contain iron-sulfur clusters, and copper ions disrupt the stability of these clusters, leading to protein degradation and functional loss. Du et al. showed that copper directly binds to the iron-sulfur cluster assembly proteins ISCA1 (iron-sulfur cluster assembly 1), ISCA2 (iron-sulfur cluster assembly 2), and ISCU (iron-sulfur cluster assembly enzyme), hindering normal iron-sulfur cluster biogenesis and consequently reducing the activity of iron-sulfur cluster-containing enzymes (Du et al., 2023). FDX1 itself contains a [2Fe-2S] cluster, and its function is highly dependent on the integrity of this cluster. Deep mutational scanning studies have confirmed that mutation of the cysteine residues responsible for coordinating the iron-sulfur cluster in FDX1 completely abolishes its ability to induce cuproptosis (Hsiao et al., 2025). This finding reveals a noteworthy positive feedback loop. Copper-induced disruption of the FDX1 iron-sulfur cluster further weakens LIAS activity, reduces DLAT lipoylation, and causes dysfunction of mitochondrial respiratory chain complexes I/II due to iron-sulfur cluster loss, further exacerbating mitochondrial damage and creating a self-amplifying process (Du et al., 2023). This positive feedback mechanism may be the molecular basis for the irreversibility of cuproptosis once initiated (Lu et al., 2025).

Although the core molecular machinery of cuproptosis (FDX1-DLAT-LIAS axis) is conserved across cell types, the brain’s unique copper metabolic environment introduces distinct regulatory features that are often overlooked in studies of peripheral tumors. Unlike most peripheral tissues, the brain maintains exceptionally high copper concentrations, with regional levels in the substantia nigra and locus coeruleus far exceeding those in the liver or kidney (Zecca et al., 2004). Furthermore, neurons and astrocytes exhibit a specialized division of labor in copper handling: neurons predominantly express ATP7A and superoxide dismutase 1, whereas astrocytes rely heavily on metallothionein for copper sequestration and detoxification (Wang et al., 2025). This cell-type-specific copper partitioning creates a heterogeneous vulnerability landscape within the CNS, where tumor cells of different origins may inherit distinct copper metabolic dependencies. These CNS-specific features suggest that cuproptosis regulation in brain tumors may deviate from the paradigms established in peripheral cancers, and that therapeutic strategies must account for the unique copper microenvironment of the brain.

### 3.2 Process of cuproptosis

The occurrence of cuproptosis can be divided into three stages, a framework that helps understand its dynamic process (Lu et al., 2025). The first stage is copper ion overload. Whether due to active delivery by copper ionophores or aberrant activation of copper uptake pathways, when the concentration of intracellular free copper ions exceeds the buffering capacity of copper chaperones, the door to cuproptosis opens (Li et al., 2025a). The second stage is mitochondrial targeting. Excess copper ions enter mitochondria, are reduced to monovalent copper ions by FDX1, and then bind to lipoylated proteins such as DLAT. Notably, this reduction step is a key rate-limiting step in cuproptosis, as monovalent copper ions have a much higher affinity for lipoylation sites than divalent copper ions. The third stage is proteotoxic stress and cell death. Aberrant DLAT aggregation impairs pyruvate dehydrogenase complex function, accompanied by extensive degradation of iron-sulfur cluster proteins, collectively triggering mitochondrial proteotoxic stress and ultimately initiating an irreversible cell death program (Shen et al., 2025). It should be emphasized that these three stages are not simply linear. Degradation of iron-sulfur cluster proteins may occur before or simultaneously with DLAT aggregation, and both have synergistic amplifying effects on mitochondrial damage. This multi-target, multi-stage characteristic distinguishes cuproptosis from death modalities that rely on a single effector molecule and explains why cuproptosis is difficult to effectively interrupt once initiated.

### 3.3 Regulators of cuproptosis sensitivity

Given that cuproptosis depends on four sequential steps, namely, copper overload, FDX1-mediated reduction, DLAT lipoylation, and iron-sulfur cluster integrity, cellular sensitivity to cuproptosis is naturally determined by the regulatory state of each node in these steps. Arranging these factors in a causal chain reveals a clear hierarchical structure.

At the most upstream level is the rate and total amount of copper entering the cell. CTR1 expression levels directly determine copper uptake flux, and cells with high CTR1 expression are more sensitive to cuproptosis (Furukawa et al., 2008). However, copper efflux is equally critical. ATP7A and ATP7B effectively reduce the concentration of copper accessible to mitochondria by pumping copper out of the cell or sequestering it into vesicles. Therefore, the CTR1 to ATP7A expression ratio may more accurately predict cuproptosis sensitivity than either single indicator alone. A noteworthy paradox is that tumor cells often upregulate CTR1 to meet metabolic demands during proliferation but simultaneously must upregulate ATP7A to prevent copper overload, placing them in a fragile steady-state balance.

Moving downstream, FDX1 expression level is the most critical intracellular determinant. This is not simply because it is a node in the chain, but because it simultaneously controls copper activation and the construction of attack targets. Cells with low or absent FDX1 expression are almost completely resistant to cuproptosis (Liu et al., 2025a). This finding has direct clinical significance. FDX1 expression levels vary significantly among different tumor types and even among different subtypes of the same tumor, providing a potential biomarker for patient stratification.

Further downstream, the extent of DLAT lipoylation determines the number of copper ion binding sites. LIAS catalyzes this modification, and its expression levels therefore indirectly regulate sensitivity (Zhao et al., 2024). However, there is a subtlety here. LIAS itself also contains iron-sulfur clusters, making it both a tool required for cuproptosis and a target of copper attack. This dual identity means that in the early stage of copper overload, LIAS activity may transiently increase, but as iron-sulfur clusters are destroyed, its function will rapidly be lost, forming a “transient increase followed by collapse dynamic process”.

The antioxidant system, particularly glutathione, acts further downstream, primarily regulating cellular tolerance to copper overload. Glutathione not only scavenges reactive oxygen species induced by copper overload but also directly chelates free copper ions (Liu et al., 202b). This means that cells with higher glutathione levels can tolerate higher degrees of copper overload without triggering cell death, without altering copper uptake or efflux. Since the glutathione system shares regulatory nodes with ferroptosis, this finding also suggests functional overlap between cuproptosis and ferroptosis (Xue et al., 2025).

At the most downstream level is the cellular metabolic state. The core attack target of cuproptosis is mitochondria, so the degree of cellular dependence on mitochondrial function directly determines the lethality of the attack. Cells highly dependent on oxidative phosphorylation will experience rapid collapse of energy metabolism once mitochondria are damaged. In contrast, cells primarily relying on glycolysis remain relatively resistant, as they can maintain basic ATP supply even with impaired mitochondrial function (Xing et al., 2023). This logical chain explains why tumor cells with a glycolytic phenotype are insensitive to cuproptosis and suggests that forced reversal of metabolic phenotype may be an effective strategy to overcome resistance.

### 3.4 Comparison and cross-talk between cuproptosis and other cell death modalities

Cuproptosis does not occur in a vacuum. Tumor cells simultaneously express multiple death pathways, which have both functional overlap and mutual constraints. Understanding these relationships is crucial for interpreting resistance mechanisms and designing combination therapies (Wei et al., 2025).

The relationship between cuproptosis and apoptosis is the simplest and most direct. The two are mechanistically completely independent. Cuproptosis does not depend on caspases and does not exhibit DNA fragmentation or plasma membrane blebbing (Guo et al., 2025b). Therefore, it is insensitive to common apoptotic resistance mechanisms such as Bcl-2 overexpression or p53 mutations. This independence has two implications. At the basic level, it means cuproptosis is a genuinely alternative death pathway. At the applied level, it means tumors resistant to apoptosis inducers may still be sensitive to cuproptosis inducers.

The relationship between cuproptosis and ferroptosis is more complex. Ferroptosis is an iron-dependent, lipid peroxidation-driven form of cell death characterized by glutathione depletion and GPX4 inactivation (Li et al., 2025b). Cuproptosis and ferroptosis share multiple regulatory nodes, including mitochondrial dysfunction, regulation by the glutathione system, and mutual influence through iron-sulfur cluster protein damage (Song et al., 2025a). Studies have shown that copper overload can induce ferroptosis by upregulating HO-1 (heme oxygenase 1) activity and increasing lipid ROS and malondialdehyde levels (Xiang et al., 2025). However, their core mechanisms differ. The key feature of ferroptosis is lipid peroxidation, while that of cuproptosis is lipoylated protein aggregation and iron-sulfur cluster destabilization (Liu and Chen, 2024). This suggests that combined use of both inducers may produce synergistic effects. Cuproptosis attacks the core of mitochondrial metabolism, while ferroptosis attacks membrane lipid integrity, and the two pathways can proceed simultaneously without interfering with each other.

The role of autophagy in cuproptosis is twofold. Elevated copper levels can induce autophagy activation (Liu et al., 2021), but autophagy simultaneously counteracts cuproptosis by clearing aberrantly aggregated DLAT protein and promoting iron-sulfur cluster protein synthesis. Hypoxia-induced autophagy can confer resistance to cuproptosis by inhibiting FDX1-mediated pathways (Qin et al., 2025). Therefore, inhibiting autophagy instead enhances cuproptosis sensitivity. This finding suggests that autophagy inhibitors may serve as sensitizers for cuproptosis-based therapy.

PANoptosis is another form of regulated cell death proposed in 2019, fundamentally distinct from cuproptosis. PANoptosis is characterized by concurrent features of pyroptosis, apoptosis, and necroptosis, coordinately regulated by the PANoptosome complex (Malireddi et al., 2019). Mechanistically, PANoptosis depends on the assembly of RIPK1 (receptor-interacting protein kinase 1), caspase-8, ZBP1 (Z-DNA binding protein 1), and ASC (apoptosis-associated speck-like protein containing a CARD) into multiprotein complexes (Li et al., 2025c), while cuproptosis depends on FDX1-mediated copper reduction and DLAT lipoylation aggregation. There is potential cross-talk in reactive oxygen species regulation, but overall, these are two parallel death pathways. This independence means that interventions targeting one pathway are unlikely to directly affect the other.

Cuproptosis itself is immunogenic, a property worth emphasizing. Cells undergoing cuproptosis activate the cGAS-STING (cyclic GMP-AMP synthase-stimulator of interferon genes) pathway, release damage-associated molecular patterns, promote dendritic cell maturation and antigen presentation (Wu et al., 2025c). Cuproptosis promotes the production of pro-inflammatory cytokines such as IFNγ (interferon-gamma) and IL-2 (interleukin-2), enhances the infiltration and activity of CD8^+^ T cells and natural killer cells (Weng et al., 2026), and induces polarization of tumor-associated macrophages toward the antitumor M1 phenotype (Yang et al., 2025b). This property provides a theoretical basis for combining cuproptosis inducers with immunotherapy, and studies have confirmed that combined application of cuproptosis inducers with anti-PD-1/PD-L1 antibodies significantly enhances antitumor efficacy (Zhang et al., 2025a).

To more intuitively illustrate the similarities and differences between cuproptosis and other major cell death modalities, Table 1 summarizes their differences in core triggers, key effector molecules, regulatory mechanisms, characteristic morphologies, immunogenicity, and major inhibitory mechanisms.

**TABLE 1:** Comparison of core features of cuproptosis, apoptosis, ferroptosis, autophagy, and PANoptosis.

Feature	Cuproptosis	Apoptosis	Ferroptosis	Autophagic cell death	PANoptosis
Core trigger	Copper overload	Death receptor/mitochondrial damage	Iron overload/GPX4 inactivation	Autophagy overactivation	Pathogen/inflammatory signals
Key effector molecules	FDX1, DLAT	Caspase family	ACSL4, lipoxygenases	ATG family, LC3	PANoptosome complex
Regulatory mechanism	Copper reduction + lipoylation aggregation	Cascade enzymatic cleavage	Lipid peroxidation	Lysosomal degradation	Simultaneous pyroptosis + apoptosis + necroptosis
Characteristic morphology	Mitochondrial contraction, membrane rupture	Cell shrinkage, nuclear condensation	Mitochondrial atrophy, increased membrane density	Autophagic vacuole formation	Mixed morphological features
Immunogenicity	High	Low/medium	High	Medium	High
Major inhibitory mechanisms	Copper efflux, FDX1 downregulation	Bcl-2 overexpression	GPX4 upregulation	ATG inhibitors	PANoptosome inhibitors
Key references	(Tsvetkov et al., 2022; Song et al., 2025b; Mao et al., 2025)	(Kal et al., 2018; Malone et al., 2025; Chipuk et al., 2010; Green et al., 2009)	(Dixon et al., 2012; Yang et al., 2014; Lei et al., 2022)	(Huang et al., 2025; Guerra-Andrés et al., 2025)	(Malireddi et al., 2019; Li et al., 2025c)

Abbreviations: FDX1, ferredoxin 1; DLAT, dihydrolipoamide S-acetyltransferase; GPX4, glutathione peroxidase 4; ACSL4, acyl-CoA, synthetase long-chain family member 4; ATG, autophagy-related; LC3, microtubule-associated protein 1A/1B-light chain 3.

In summary, cuproptosis is mechanistically completely independent of apoptosis, explaining its ability to bypass apoptotic resistance. It shares regulatory nodes with ferroptosis but has a distinct core mechanism, providing opportunities for combined targeting. It is antagonized by autophagy, suggesting that autophagy inhibition may serve as a sensitization strategy. It possesses immunogenicity, laying the foundation for combination with immunotherapy. These relationships will be further elaborated in Chapter 6 regarding therapeutic strategies.

## 4 Research progress on cuproptosis in central nervous system tumors

### 4.1 Glioma

As discussed earlier, glioma cells exist in a state of “copper tension.” They actively increase copper uptake by upregulating CTR1 to meet proliferative demands while simultaneously upregulating ATP7A to prevent copper overload. This steady-state balance is both a prerequisite for glioma survival and a source of vulnerability. The question is whether this “copper tension” state indeed translates into sensitivity to cuproptosis inducers. Research in this area is ongoing.

Glioma is the most common primary malignant tumor of the central nervous system, accounting for approximately 80% of all malignant brain tumors (Zhang et al., 2025b; Weller et al., 2024). According to the WHO grading system, gliomas are classified into grades 1 to 4, with glioblastoma (WHO grade 4) being the most aggressive subtype, with a median survival of only 9–18 months (Piyadasa et al., 2026). Standard treatment for glioma includes maximal safe resection, radiotherapy, and temozolomide chemotherapy (Sipos et al., 2025). However, due to tumor heterogeneity, the presence of glioma stem cells, and blood-brain barrier limitations, recurrence is almost inevitable (He et al., 2025). This dilemma has prompted researchers to re-examine the metabolic vulnerabilities of gliomas, and aberrant copper metabolism provides a new entry point.

Multiple studies have consistently shown that copper levels in the serum and tumor tissues of glioma patients are significantly higher than those in healthy individuals (Gao et al., 2025), and copper concentrations increase with tumor malignancy grade (Zhang et al., 2024). More importantly, glioma cells with high CTR1 expression are more sensitive to copper ionophores, and CTR1 knockdown reduces sensitivity (Wang et al., 2023). This means that the “high copper uptake” state actively established by glioma cells for proliferation precisely serves as an entry point for cuproptosis inducers.

FDX1 plays an executing role in this logical chain. FDX1 is highly expressed in glioma and is directly regulated by NFKB1 (Wu et al., 2025a). Glioma cells with high FDX1 expression are more sensitive to elesclomol, and knockdown significantly reduces sensitivity. This presents a seemingly paradoxical but actually consistent phenomenon. High FDX1 expression both promotes glioma proliferation and invasion and enhances cuproptosis sensitivity. This “double-edged sword” characteristic precisely constitutes the theoretical basis for targeted therapy. Cuproptosis inducers convert molecules that originally promote tumor progression into weapons that kill tumors.

However, the above logic mainly applies to more differentiated glioma cells. The presence of glioma stem cells complicates the issue. Yuan et al. found that glioma stem cells exhibit relative resistance to cuproptosis, and this resistance is closely related to the copper efflux protein ATP7A (Yuan et al., 2026). A noteworthy finding is that ATP7A expression is regulated by the circadian core transcription factor BMAL1 (brain and muscle ARNT-like 1), resulting in circadian rhythmicity of copper homeostasis in glioma stem cells (Yuan et al., 2026). This suggests that the timing of cuproptosis inducer administration may affect therapeutic efficacy. Dosing during periods of low BMAL1 expression and low ATP7A levels might more effectively kill glioma stem cells. Furthermore, copper levels themselves form a negative feedback loop through p62-mediated lysosomal degradation of BMAL1, maintaining circadian rhythmicity. Drugs targeting BMAL1 or ATP7A, such as omeprazole, have shown synergistic effects with copper ionophores in preclinical studies (Gu et al., 2025).

Based on the copper metabolism features of glioma, potentially effective therapeutic strategies can be proposed. First, target more differentiated tumor cells by exploiting the cuproptosis sensitivity conferred by high CTR1 and FDX1 expression to directly induce cuproptosis. Second, target glioma stem cells by inhibiting ATP7A or targeting BMAL1 to overcome their cuproptosis resistance. Combining both strategies may achieve tumor killing and prevent recurrence.

### 4.2 Medulloblastoma

If the cuproptosis sensitivity of gliomas originates from adaptive reprogramming of copper metabolic pathways, then the sensitivity of medulloblastoma comes from a different molecular route. Medulloblastoma is the most common malignant brain tumor in children (Liu et al., 2025b).

Medulloblastoma is molecularly classified into four subtypes: WNT-activated, SHH-activated, Group 3, and Group 4 (Kiang et al., 2025). Among these four molecular subtypes, Group 3 is the most aggressive, frequently associated with c-MYC amplification and the poorest prognosis (Liu et al., 2025b). Approximately 30% of patients experience tumor recurrence (Peterson et al., 2024), and radiotherapy carries significant risks of cognitive impairment in the developing brain, further limiting treatment options (Mayr et al., 2026).

Dang et al. performed metabolomic analysis and found that Group 3 medulloblastoma highly expresses DLAT, with expression directly regulated by c-MYC (Dang et al., 2025). The logical chain of this finding is clear. c-MYC amplification drives DLAT high expression, and DLAT is the core target for copper binding and cuproptosis initiation. Therefore, c-MYC-driven tumors are highly sensitive to elesclomol-induced cuproptosis. IDH1 (isocitrate dehydrogenase 1) regulates DLAT expression through c-MYC, creating a targetable metabolic vulnerability (Dang et al., 2025). In glioma research, elesclomol achieved efficient blood-brain barrier penetration through RAP-mediated targeting (Liu et al., 2025c), a strategy equally applicable to medulloblastoma. This agent has now entered preclinical development.

Notably, the mechanism of cuproptosis sensitivity in medulloblastoma differs fundamentally from that in glioma. Glioma sensitivity derives from upregulation of the copper uptake system, while medulloblastoma sensitivity derives from upregulation of downstream effector molecules such as DLAT. This means the two tumor types may respond differently to different types of cuproptosis inducers. Drugs targeting copper uptake may be more effective in gliomas with high CTR1 expression, while drugs directly acting on DLAT may have greater advantages in medulloblastoma. This difference suggests that future clinical studies need to select drugs individually based on tumor molecular characteristics.

### 4.3 Pituitary neuroendocrine tumors

Pituitary neuroendocrine tumors are common intracranial tumors originating from anterior pituitary neuroendocrine cells, previously termed pituitary adenomas (Villa et al., 2025). According to the 2022 WHO classification standards, these tumors have been renamed pituitary neuroendocrine tumors to more accurately reflect their neuroendocrine cell origin and biological characteristics (Asa et al., 2022). Pituitary neuroendocrine tumors account for approximately 15%–20% of all intracranial tumors, making them the second most common primary intracranial tumor after meningioma (Klit et al., 2025). Most are benign, but invasive tumors frequently invade critical structures such as the cavernous sinus, leading to high postoperative residual rates and recurrence (Casar-Borota et al., 2025). Approximately 20%–30% of prolactinomas are resistant to dopamine receptor agonists, and for progressive tumors resistant or intolerant to temozolomide, no standard subsequent treatment options currently exist (Casar-Borota et al., 2025).

The emergence of cuproptosis introduces a new variable to this dilemma. Huang et al. performed high-throughput drug screening on 17 patient-derived primary pituitary neuroendocrine tumor cells and found that multiple copper ionophores, including disulfiram, elesclomol, and zinc pyrithione, effectively inhibited tumor cell growth (Huang et al., 2024a). The value of this study lies not in discovering a new drug but in providing a complete chain of evidence. The cytotoxic effects in primary cells ruled out cell line-specific false positives, mechanistic validation ruled out nonspecific toxicity, and animal experiments ruled out *in vitro* artifacts. Through this stepwise progression of three layers of evidence, the study reached a relatively reliable conclusion: pituitary neuroendocrine tumors resistant to conventional therapy may be sensitive to cuproptosis inducers.

However, this study also has obvious limitations. The sample size was only 17 cases, and no stratification analysis was performed for different molecular subtypes of pituitary neuroendocrine tumors. Tumors of the PIT1 (pituitary-specific transcription factor 1), TPIT (T-box transcription factor), and SF1 (steroidogenic factor 1) lineages may have substantial differences in metabolic features, which could affect their sensitivity to cuproptosis. Furthermore, the long-term safety and acquired resistance mechanisms of cuproptosis inducers in pituitary neuroendocrine tumors remain unclear. Future studies need to validate these findings in larger patient cohorts and systematically evaluate combination regimens of cuproptosis inducers with existing treatment strategies.

### 4.4 Meningioma

Meningioma is a common primary tumor of the central nervous system (Huang et al., 2024a). However, WHO grade 2 and grade 3 high-grade subtypes have stronger invasiveness and higher recurrence risk and respond much more poorly to conventional surgery and radiotherapy (Okano et al., 2022). The clinical management of these patients represents a true challenge.

Research on copper metabolism in meningioma presents a noteworthy contradiction. Kurisaka et al. found that expression of copper-zinc superoxide dismutase was significantly higher in more malignant meningioma subtypes than in low-grade subtypes (Kurisaka and Mori, 1993). In contrast, Yoshida et al. found that total tissue copper content in meningioma was not significantly different from normal controls and was much lower than that in malignant glioma (Yoshida et al., 1993). These two studies appear contradictory but actually reveal different dimensions of the same issue. Copper-related protein expression levels do not always parallel total tissue copper content, which may reflect fundamental differences in copper subcellular distribution or binding status between meningioma and glioma.

Direct evidence for cuproptosis in meningioma comes from the systematic work of Kao et al. Disulfiram combined with copper significantly inhibited the viability of adherent meningioma cells and meningioma stem-like cells, and this cytotoxicity was strictly copper-dependent (Kao et al., 2022).

Mechanistic studies revealed that disulfiram combined with copper increases intracellular copper levels, induces cellular senescence, endoplasmic reticulum stress, and apoptosis, and inhibits the expression of multiple aldehyde dehydrogenase isoforms. Notably, ALDH (aldehyde dehydrogenase) is an important marker of cancer stem cells, and its inhibition by disulfiram combined with copper suggests the possibility of suppressing tumor regeneration at its source.

This finding has important theoretical implications. Meningioma has low baseline copper content, yet exogenous copper overload can still effectively induce cell death. This means that the antitumor effect of cuproptosis inducers does not depend on endogenous copper levels but on the cellular response capacity to copper overload. Even though the baseline copper content of meningioma is lower than that of glioma, exogenous administration of copper ionophores can still overcome this limitation and achieve effective therapeutic concentrations. Currently, this field still lacks validation in vivo animal models. Whether disulfiram combined with copper can effectively cross the blood-brain barrier and reach effective concentrations in intracranial meningioma tissue remains to be determined.

To provide an integrated overview of the cuproptosis features across the four CNS tumor types discussed above, Figure 2 summarizes the key molecular characteristics and sensitivity profiles of glioma, medulloblastoma, pituitary neuroendocrine tumors, and meningioma.

**FIGURE 2:**
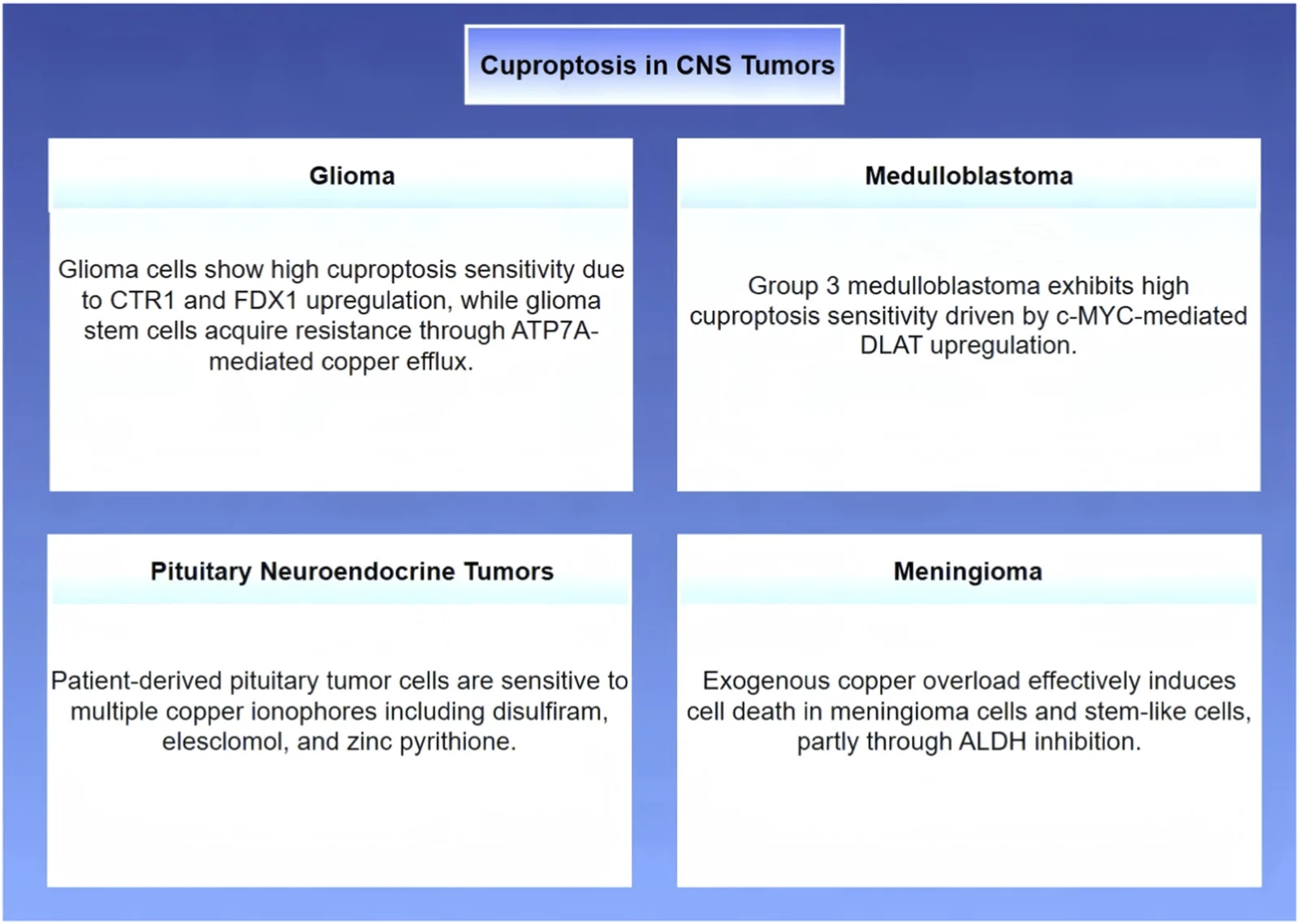
Summary of cuproptosis in four major types of central nervous system tumors. Four panels summarize the key molecular features of glioma, medulloblastoma, pituitary neuroendocrine tumors, and meningioma. Abbreviations: CNS, central nervous system; CTR1, copper transporter 1; FDX1, ferredoxin 1; ATP7A, ATPase copper transporting alpha; c-MYC, cellular myelocytomatosis oncogene; DLAT, dihydrolipoamide S-acetyltransferase; ALDH, aldehyde dehydrogenase.

### 4.5 The role of cuproptosis in radiation-induced brain injury

Radiation-induced brain injury is one of the most serious complications following intracranial radiotherapy for head and neck cancer patients, and effective treatments are currently lacking. Radiation induces intracellular reactive oxygen species bursts, and reactive oxygen species can directly attack mitochondrial iron-sulfur cluster proteins, a mechanism highly overlapping with cuproptosis (Forenzo and Larsen, 2024). Wu et al. used spatial transcriptomics to validate this hypothesis for the first time, demonstrating that neuronal cuproptosis indeed plays a critical role in radiation-induced brain injury (Wu et al., 2026).

The researchers constructed a biomimetic delivery system loaded with UK5099, a mitochondrial pyruvate uptake inhibitor that effectively inhibits cuproptosis, and melatonin, which scavenges reactive oxygen species, inhibits excessive glial cell activation, and downregulates pro-inflammatory cytokines, providing multiple neuroprotective effects (Wu et al., 2026). This system, delivered via the nasal-brain route, rapidly distributes to injured brain tissue, significantly improves behavioral function in radiation-induced brain injury mice, reduces cerebral edema, and increases neuronal survival, with efficacy superior to either cuproptosis-targeting or neuroinflammation-targeting monotherapy.

The evidence chain of this study is complete. First, spatial transcriptomics identified changes in cuproptosis-related gene expression in radiation-injured brain tissue, establishing an association between cuproptosis and radiation-induced brain injury. Second, the use of the cuproptosis inhibitor UK5099 validated the pathological significance of this pathway. Finally, dual-targeting intervention simultaneously modulating cuproptosis and neuroinflammation with melatonin demonstrated superiority. This study not only reveals the pathological role of cuproptosis in radiation-induced brain injury but, more importantly, provides a paradigm for the application of cuproptosis inhibitors in central nervous system diseases.

This study has significance at two levels. Pathologically, it establishes for the first time a link between cuproptosis and radiation-induced brain injury, revealing a previously overlooked mechanism. Therapeutically, it provides a paradigm for the application of cuproptosis inhibitors in central nervous system diseases. Copper overload can impair cognitive function through multiple pathways including inducing neuroinflammation, oxidative stress, mitochondrial dysfunction, and cuproptosis (Zhu Z. et al., 2024), and this mechanism may also play important roles in neurodegenerative diseases such as Alzheimer’s disease and Parkinson’s disease. Therefore, cuproptosis-targeted therapeutic strategies may extend to a broader range of neurological disorders.

## 5 Cuproptosis-targeted therapeutic strategies

Based on the systematic review of cuproptosis molecular mechanisms above, cuproptosis-targeted therapeutic strategies have evolved from single small-molecule copper ionophores to copper-based nanomaterials and combination therapies. These three types of strategies follow a clear evolutionary logic: from directly targeting core molecules to solving delivery bottlenecks, and then to overcoming resistance through mechanistic synergy. Copper ionophores directly deliver copper ions to mitochondria to trigger core pathways. On this basis, copper-based nanomaterials address blood-brain barrier delivery and enable multimodal therapy. Combination therapies further cover tumor heterogeneity and resistance escape pathways through synergistic effects. The three are not alternatives but complementary approaches for different clinical problems.

### 5.1 Copper ionophores

Copper ionophores are the most extensively studied class, with the most direct design logic. They utilize the high affinity of molecules for copper ions to efficiently deliver copper into cells to achieve copper overload. The two most thoroughly studied carriers are elesclomol and disulfiram.

Elesclomol is the first copper ionophore to enter clinical trials, with advantages reflected in two key characteristics. First, it selectively delivers copper to mitochondria after forming a complex with divalent copper ions, showing significant antitumor activity in glioma and medulloblastoma models with good blood-brain barrier penetration (Tarin et al., 2023). Second, the transmembrane transport of its copper complex is not completely dependent on the CTR1 channel (Chen et al., 2025b), meaning that for tumor cells with reduced copper uptake due to SLC31A1 (solute carrier family 31 member 1) downregulation, elesclomol may retain partial activity.

Disulfiram represents another route with greater translational advantages. As an FDA-approved drug for alcohol dependence, its over 60 years of clinical use and well-established safety record significantly lower the barriers to new indication development. Mechanistically, the active metabolite of disulfiram, diethyldithiocarbamate, forms a complex with copper and induces cuproptosis through the FDX1-DLAT axis, sharing the same downstream effector pathway as elesclomol (Kang et al., 2023). The differences in delivery methods and pharmacokinetics between the two determine their applicability in different clinical scenarios. Currently, multiple clinical trials of disulfiram combined with copper supplements for glioma are ongoing or have completed preliminary reports, representing the direction closest to clinical translation in the cuproptosis field. Several clinical trials of disulfiram combined with copper for glioma have been initiated. A phase II trial evaluated disulfiram/copper in recurrent glioblastoma and reported low toxicity but no objective responses, with only a minority of patients achieving clinical benefit (Huang et al., 2019). A randomized phase II/III trial comparing disulfiram/copper add-on to alkylating chemotherapy versus chemotherapy alone in recurrent glioblastoma found no significant difference in 6-month survival, while grade ≥3 adverse events were significantly higher in the disulfiram group (Werlenius et al., 2023). A phase I/II trial combining disulfiram/copper with concurrent radiation and temozolomide in newly diagnosed glioblastoma reported manageable toxicity and showed preliminary efficacy (Huang et al., 2024b). Collectively, these trials suggest that disulfiram/copper has limited benefit in unselected glioblastoma populations, but specific molecular subtypes (e.g., BRAF-mutant) may derive greater benefit, and the combination remains an active area of investigation.

### 5.2 Copper-based nanomaterials

The emergence of copper-based nanomaterials addresses the limitations of small-molecule copper ionophores, such as narrow pharmacokinetic windows and insufficient intratumoral accumulation. Their core logic is to use nanoscale structural design to address blood-brain barrier delivery while achieving responsive release and multimodal therapy.

This field can be divided into two technical paths. The first is copper metal-organic frameworks, focusing on active regulation of copper homeostasis. Zhang et al. developed a TP0751 peptide-modified stem cell membrane-wrapped copper-based metal-organic framework delivery system integrating two functions. TP0751 peptide mediates blood-brain barrier transcytosis while delivering small interfering RNA to silence ATP7A and block copper efflux. The deep logic of this design is that simply increasing copper intake may be offset by cellular efflux mechanisms, and only simultaneous inhibition of efflux can truly disrupt copper homeostasis (Zhang et al., 2023).

The second is copper-doped nanomaterials, focusing on coupling cuproptosis with other therapeutic modalities. Cheng et al. synthesized copper-doped carbon dots as sonosensitizers. Copper doping transforms carbon dots into p-n type semiconductors, narrowing the band gap and prolonging electron-hole separation lifetime, thereby greatly improving the efficiency of reactive oxygen species generation under ultrasound excitation. Simultaneously, this material directly induces cuproptosis by upregulating DLAT expression, integrating sonodynamic therapy and cuproptosis into a single nanoplatform (Cheng et al., 2024). Zhu et al. constructed carrier-free self-assembling nanoparticles Ce6@Cu NPs, which generate reactive oxygen species and consume glutathione through sonodynamic effects to induce ferroptosis, while monovalent copper ions induce lipoylated DLAT oligomerization to trigger cuproptosis, with the two pathways mutually amplifying through mitochondrial reactive oxygen species positive feedback (Zhu et al., 2024c). The common logic of these studies is that both cuproptosis and ferroptosis target mitochondria as core targets, with mechanistic overlap, and simultaneous activation can produce synergistic killing effects.

Building on the above nanomaterials, researchers have further developed active targeting delivery systems that achieve higher precision drug delivery through ligand-receptor recognition. Compared with nanomaterials relying on passive targeting or microenvironment responsiveness, the core advantage of active targeting systems is stronger specificity. Liu et al. developed RAP-modified liposomes. The RAP peptide binds to low-density lipoprotein receptor-related protein 1, which is highly expressed on blood-brain barrier endothelial cells and glioma cell surfaces. This system not only efficiently crosses the blood-brain barrier but, more importantly, triggers FDX1-dependent cuproptosis by upregulating FDX1 expression, inducing DLAT aggregation, and depleting iron-sulfur cluster proteins, coupling targeted delivery with activation of the core cuproptosis pathway (Liu et al., 2025c). Ding et al. designed targeting ligand-modified nanodiscs co-delivering carfilzomib and disulfiram-copper complex, simultaneously inducing two completely different death modalities, apoptosis and cuproptosis, covering a broader population of tumor cells (Ding et al., 2025).

### 5.3 Combination therapy strategies

Combination therapy strategies push the above logic to a deeper level. The most translationally promising direction of cuproptosis lies not in its single-agent killing effect but in its immunogenic cell death characteristics. Tumor cells undergoing cuproptosis release damage-associated molecular patterns such as HMGB1 (high mobility group box 1), ATP, and CRT (calreticulin), activate dendritic cell maturation and antigen presentation, and subsequently recruit CD8^+^ T cells and natural killer cells through the cGAS-STING pathway. This characteristic makes cuproptosis an ideal partner for immunotherapy. Cuproptosis inducers not only directly kill tumor cells but also remodel the tumor immune microenvironment, converting “cold tumors” to “hot tumors,” thereby enhancing the efficacy of immune checkpoint inhibitors (Cui et al., 2026).

Along this logic, Chen et al. constructed a copper coordination-driven brain-targeting nano-assembly that dual-activates the cGAS-STING pathway through photodynamic therapy and cuproptosis while downregulating PD-L1 (programmed death-ligand 1) expression. Combined with anti-PD-L1 antibodies, it significantly enhanced antitumor immunity, forming a positive cycle of “inducing death-activating immunity-eliminating tumors” (Chen et al., 2024). Jiang et al. combined cuproptosis with radiotherapy, constructing nanoliposomes co-delivering elesclomol and copper peroxide. They enhanced radiotherapy efficacy through mitochondria-targeted cuproptosis, with the logical basis that both radiotherapy and cuproptosis induce DNA damage and reactive oxygen species production, and cuproptosis-mediated mitochondrial attack can deplete the energy reserves of tumor cells, making them more sensitive to radiotherapy-induced damage (Jiang et al., 2025). Yin et al. developed a copper-deposited metal-phenolic network that generates reactive oxygen species and consumes glutathione through Fenton reactions, synergistically activating cuproptosis and ferroptosis, with the two pathways forming positive feedback through mitochondrial reactive oxygen species, effectively addressing resistance that emerges when single pathways are blocked (Yin et al., 2025).

From immunotherapy to radiotherapy to ferroptosis, the above combination strategies follow the same core logic. Cuproptosis acts as an “amplifier” or “sensitizer,” forming mechanistic synergy with other treatment modalities. The choice among the three types of combination strategies depends on the specific clinical scenario. Immunosuppressive microenvironments should prioritize combination with immune checkpoint inhibitors. Tumors with high local control requirements may benefit more from radiotherapy combinations. Multiply resistant tumors may achieve more thorough killing effects by simultaneously activating cuproptosis and ferroptosis.

Returning to the logic at the beginning of this chapter, the three types of strategies address the core problems facing cuproptosis targeting from different angles. Copper ionophores directly act on molecular targets, representing the most mechanism-clearly defined and clinically translatable strategy. Copper-based nanomaterials address delivery bottlenecks through engineering approaches while enabling multimodal therapy. Combination therapy strategies cover tumor heterogeneity and resistance escape pathways through mechanistic synergy. The three strategies are not mutually exclusive. In specific applications, they need to be combined and optimized according to tumor type, resistance mechanism, and treatment goals. For example, for copper uptake reduction-type resistance caused by CTR1 downregulation, elesclomol and other copper ionophores that do not depend on CTR1 should be prioritized. For cases with an intact blood-brain barrier, nanodelivery systems should be considered. For patients who have developed resistance to immunotherapy, combining cuproptosis inducers with immune checkpoint inhibitors may be a direction worth trying.

## 6 Discussion and perspectives

The discovery of cuproptosis has opened a new dimension for treating central nervous system tumors by targeting mitochondrial metabolism. Mechanistically, the elucidation of the FDX1-DLAT-LIAS core axis has transformed cuproptosis from a phenomenological description into a manipulable, intervenable molecular event. This mechanism does not rely on classical apoptotic pathways, giving it a unique advantage in overcoming conventional chemotherapy resistance. However, the response of central nervous system tumors such as gliomas to cuproptosis is not uniformly sensitive. Glioma stem cells exhibit significant cuproptosis resistance through ATP7A-mediated copper efflux and BMAL1-regulated circadian adaptation, meaning that clinical treatment needs to target both differentiated tumor cells and stem cell populations simultaneously to achieve durable efficacy. Meanwhile, tumor cells can acquire cuproptosis resistance through multiple pathways including reduced copper uptake, epigenetic silencing of FDX1/DLAT, activation of the glutathione system, and metabolic reprogramming toward a glycolytic phenotype. These resistance mechanisms often coexist, suggesting that it is unlikely to solve the resistance problem with a single sensitization strategy in clinical practice; rather, comprehensive approaches combining targeting of copper efflux, epigenetic regulation, and metabolic intervention are needed.

From the therapeutic strategy perspective, copper ionophores, copper-based nanomaterials, and combination therapies all show promising prospects. Elesclomol and disulfiram, as already marketed or previously clinically tested drugs, have favorable safety data and clinical translation convenience. Copper-based nanomaterials can address blood-brain barrier delivery while enabling responsive release and multimodal therapy. Particularly noteworthy is that cuproptosis is an immunogenic form of cell death that activates the cGAS-STING pathway and recruits CD8^+^ T cells, a characteristic that has produced significant synergistic effects when combined with immune checkpoint inhibitors, offering new approaches to overcome the immunosuppressive microenvironment of glioma.

Despite rapid progress, the clinical translation of cuproptosis in central nervous system tumors faces several interconnected core challenges. Normal neuron protection is paramount. The high sensitivity of the central nervous system to copper homeostasis is a double-edged sword. It remains unclear whether the difference in cuproptosis sensitivity between glioma cells and normal neurons is sufficient to constitute a safety window. Future studies need to evaluate potential neurotoxicity using co-culture systems and brain organoid models. The issue of blood-brain barrier delivery efficiency is equally challenging. Although elesclomol and disulfiram have some blood-brain barrier permeability, their brain exposure is far from sufficient to cover all tumor cells. While nanodelivery systems perform excellently in animal models, they face engineering challenges in clinical translation, including large-scale preparation and *in vivo* stability. The dynamic evolution of resistance mechanisms also cannot be ignored. Currently, little is known about the clonal evolution patterns of long-term acquired resistance, and the non-classical resistance mediated by the circadian regulatory axis in glioma stem cells deserves particular in-depth exploration. Furthermore, the lack of biomarkers makes patient stratification and treatment monitoring difficult. Although expression levels of FDX1, DLAT, and SLC31A1 have been proposed as predictive indicators, none have been prospectively validated in multicenter clinical cohorts.

Biomarker development represents another critical bottleneck for clinical translation, as no cuproptosis-related biomarker has been prospectively validated in CNS tumors to date. Based on the mechanisms discussed above, candidate biomarkers can be categorized into three functional classes. Diagnostic biomarkers may include tissue and CSF copper levels. Predictive biomarkers could include CTR1, FDX1, DLAT, and the CTR1/ATP7A ratio. Prognostic biomarkers may include FDX1 and DLAT expression, which have been associated with survival in glioma and medulloblastoma (Lu et al., 2022; Zhou et al., 2024). Beyond tissue-based markers, liquid biopsy strategies offer non-invasive approaches for dynamic monitoring. CSF copper levels may reflect the copper metabolic status of CNS tumors. Exosomal cuproptosis-related molecules, including FDX1, DLAT, and copper chaperones, represent another emerging modality, as tumor-derived exosomes can cross the blood-brain barrier into CSF and circulation. The combination of multiple biomarkers may improve predictive accuracy. However, the clinical utility of these candidates requires rigorous validation through prospective studies with standardized protocols.

Looking forward, the translational path of cuproptosis in central nervous system tumors may need to proceed along three directions simultaneously. At the basic level, single-cell multi-omics and spatial transcriptomics should be used to dissect the heterogeneous responses of different cells in the tumor microenvironment to cuproptosis, and to deeply explore the interaction mechanisms between cuproptosis and other death modalities such as ferroptosis and autophagy. At the technological level, smarter responsive nanosystems such as pH-responsive, reactive oxygen species-responsive, or enzyme-responsive systems should be developed to achieve precise intratumoral copper release, while optimizing delivery strategies including receptor-mediated transcytosis, nasal administration, and focused ultrasound blood-brain barrier opening. At the clinical level, priority should be given to repurposed drugs with well-established safety profiles such as disulfiram for early signal-seeking trials, simultaneously establishing patient stratification systems based on cuproptosis core molecules, and using cerebrospinal fluid liquid biopsy as an exploratory strategy for dynamic monitoring of treatment responses.

In summary, cuproptosis not only expands our understanding of the diversity of cell death but also provides a new paradigm of metabolic targetability for central nervous system tumors, a field that has long lacked effective targeted strategies. Integrating interdisciplinary perspectives from copper biology, nanotechnology, and tumor immuno-oncology holds promise for ultimately translating cuproptosis into a meaningful tool in neuro-oncology clinical practice.
